# Passing Strategies and Performative Identities: Coping with (In)Visible Chronic Diseases

**DOI:** 10.1007/s10912-019-09600-x

**Published:** 2019-12-20

**Authors:** Tanisha Jemma Rose Spratt

**Affiliations:** Department of Sociology, University of Cambridge, 16 Mill Lane, Cambridge, CB2 1SB UK

**Keywords:** Passing, Body image, Identity, Race, Embodiment, Chronic illness

## Abstract

In this article I consider the role of passing and performance in the everyday lives of alkaptonuria (AKU) and vitiligo patients. Race, LGBTQ, gender and disability scholars have long used the term passing to describe sub-groups of people within marginal populations who intentionally manipulate their bodies or alter their behaviour in order to claim identities that are not socially assigned to them at birth. In this paper I demonstrate the effectiveness of the passing strategies that patients use in order to mitigate their disease symptoms and render them invisible, thus enabling them to pass as “healthy” or unaffected by their condition. I further consider how patients who choose not to pass utilise resistance strategies in order to generate awareness of their disease and encourage funding for it. I conclude by assessing the effectiveness of these strategies in determining whether or not patients can pass, and the ways in which this is aided or hindered by their social and economic status.

## Introduction

Race, LGBTQ+, gender and disability scholars have long used the term passing to describe people from marginalised groups who intentionally change or manipulate their bodies or behaviour in order to claim identities that are not socially assigned to them at birth (Woodward 2015; Afshar 2012). Typically, these identities belong to dominant social, racial or political groups that are afforded greater privileges than those from marginalised backgrounds due to their relatively higher social, political or economic status (Walker 2001). After they alter their appearance or behaviour, passers are rendered ‘invisible’ in relation to the identities that they once claimed. Black people who pass as white are no longer seen as black, gay men and women who pass as heterosexual are no longer viewed as gay, and people with disabilities who pass as able-bodied are no longer understood to be physically or socially limited (Dawkins 2012; Persson and Richards 2008; Samuels 2003). Passing destabilises identity categories by challenging the assumption that people who look or behave a certain way belong to specific racial, class or gender groups. Because identity categories are socially and politically constructed (Dashtipour 2012), they are subject to the shifting contexts in which they are formed and given meaning. What might be considered “feminine” or “masculine” in one context is not necessarily so in another, and one can be simultaneously white and black in different contexts depending on how each racial category is locally defined.^1^ As argued by Judith Butler in her work on vulnerability and lived experiences, social identities are performative and, thus, exist separately from, and pre-date, the person who enacts or claims them (2016, 24).

By having physical or behavioural attributes that are invariably understood to be “uncharacteristic” of the groups to which they socially “belong,” passers destabilise identity categories that are frequently regarded as fixed or static (Brubaker 2015). In addition, passers often arouse suspicion in those who are unable to place the passer within the confines of normative identity categories.^2^ As noted by Erving Goffman in his work on stigma, people who are able-bodied “expect the cripple to be crippled; to be disabled and helpless: to be inferior to themselves, and they will become suspicious and insecure if the cripple falls short of these expectations” (1990, 110). When a person is ill, we expect to see “evidence” of their illness. If they have the flu we anticipate that their nose will be red and that they will cough violently, and if they have food poisoning we expect that they will vomit consistently throughout the day. Even when that person’s illness cannot be seen, we presume that the body or behaviour will “show evidence” of it. Because of this, people with chronic diseases who have learned to manage their daily experiences of pain and fatigue often feel the need to “perform” their illness in a way that is directly at odds with their experience of it. They often view this as necessary in order to make their illness and/or suffering known to those who are unable to see it and, thus, have no way of understanding their experience of it.^3^

Passing can be both intentional and inadvertent. Whilst it is possible to purposefully alter one’s body in order to fit with normative bodily ideals (Bordo 2003; Davis 2003), it is also possible to inadvertently pass as a member of a “deviant” or stigmatised group without making any bodily alterations. As noted by Sara Ahmed in her work on racial passing, “passing may function at the level of the intentional subject (the subject who seeks to pass in order to secure something otherwise unavailable to them), or it may function as a misrecognition on the part of others (one may pass for something other than one’s self-identification but not seek to, or know it)” (1999, 92). This paper will discuss passing by considering the extent to which illness is performed or enacted by chronic disease “patients” who seek to either pass as “healthy” or make their disease known.^4^ In doing so it will use two chronic diseases as case studies, alkaptonuria (AKU) and vitiligo, in order to examine the extent to which the act of passing is further complicated by people with highly visible and largely invisible chronic diseases.

AKU is a rare genetic disease that causes the patient’s bones and joint cartilage to turn black but does not typically cause any outward visible symptoms. Whilst patients often experience dark sweat and urine, both are relatively easy to conceal and, thus, largely go unnoticed. Vitiligo is an autoimmune disease of unknown origin that causes a loss of pigmentation on different areas of that person’s body. Whilst it does not negatively affect that person’s physical health, this disease can be psychologically devastating for those who witness their appearance changing (often rapidly) and are unable to do anything to prevent it.^5^ These diseases offer a useful site of comparison by collectively outlining the significance of disease visibility in relation to the practical implementation of various passing strategies. Because AKU is largely invisible, patients often feel able to pass as “healthy” despite having this condition from birth, particularly before the onset of debilitating symptoms such as joint and bone deterioration.^6^ Vitiligo, however, is often immediately visible to onlookers and often becomes more visible over time as the disease progresses. Because of this, people who have it often face numerous practical challenges in seeking to pass as “healthy.” As I will later discuss, one of the primary ways in which people with vitiligo seek to pass is by applying make up to cover their depigmented areas of skin. When those who choose to do so use these products to cover affected areas of skin that are not on their face (i.e. their hands), they often transfer on to garments and discolour them, thus clearly showing that they have attempted to conceal their condition.

When considering why people choose to pass and the different strategies that they employ in order to do so it is important to consider *who* is most likely to pass. In both disease groups the likelihood of the “patients” passing is largely determined by their social and economic status. Together, both facilitate passing by providing “patients” with the necessary resources to conceal or effectively manage their disease symptoms in a way that renders them “invisible.”^7^ Currently, the only known drug to improve common AKU symptoms is Nitisinone, which, in the United States, is only available to patients who have access to the social and economic resources that are required to obtain it. At the time of writing, the Food and Drug Administration (FDA) has not licensed Nitisinone for use by AKU patients; therefore most medical insurers do not cover the costs of this drug, and doctors are often reluctant to sign a prescription for it. Patients who do not have the financial means to pay for their Nitisinone prescription themselves and/ or who do not personally know of a doctor who is willing to sign a prescription for it cannot gain access to this drug and, as a result, have a worse overall disease experience.^8^ In a similar way, US medical insurers invariably do not cover the costs of effective vitiligo treatments that have consistently shown positive results. Because vitiligo does not negatively affect that person’s physical health it is medically recognised as a “cosmetic” disease and is thus largely viewed as a “non-medical issue” that can and should be individually managed by the people who have it. The monetary and governmental resources that would be spent in helping these people would, many argue, be better spent treating conditions that increase incidences of morbidity, such as cancer (Kaiser 2010).

As previously stated, much of the existing literature on passing assesses this phenomenon in relation to racial and gender identity as well as disability status and sexual orientation (Hobbs 2014; Fordham 1993; Siebers 2004; Woodward 2015). Whilst each of these sub-fields inform discussions of passing from an illness/ disease perspective, they do not directly address the particularities of this form of passing. Depending on their symptoms and disease stage, patients can experience flare-ups that render their disease highly visible and largely invisible at various moments. During moments when it is highly visible, passing is considerably more difficult for patients as their symptoms are harder to conceal. In addition, rather than passing for a member of a specific group that coincides with normative identity categories (i.e. white, male, able-bodied, heterosexual etc.), chronic disease patients attempt to pass as “healthy.” Because “healthiness” is a subjective ideal that is largely determined by social and cultural determinations of what that term means, there is no unifying conceptualisation of what it means to be “healthy.” Therefore, by attempting to pass as “healthy,” patients aspire to achieve their own subjective understanding of what that term means (Cornelis, Cauberghe and De Pelsmacker 2014). In this paper I will assess the effectiveness of the passing strategies that AKU and vitiligo participants reported using during the semi-structured in-depth interviews that I conducted in 2016 and 2017. I will further consider how participants who chose not to pass utilised resistance strategies in order to generate public awareness of their disease and encourage funding for it. I will conclude by evaluating the effectiveness of these strategies in determining whether or not patients are able to pass and the extent to which this is aided or hindered by their social and economic status.

## Methods

This qualitative study used semi-structured in-depth interviews with thirty-four US patients who had, at the time of the interview, been diagnosed with AKU or vitiligo by a US-based medical doctor or dermatologist. Each interview lasted between fifty minutes and three hours, and all of the patients were from the US, with the majority of them residing in the US at the time of the interview.^9^ Because AKU is a rare disease (estimated to affect roughly 1 in 250,000 – 500,000 people), I was unable to locate my sample within a specific city or region and had to include a range of people with this disease from different regions within the U.S. Because my sample with located in different regions, I was unable to meet with some of the participants face-to-face and, instead, conducted Skype and telephone interviews. I found these mediums to be largely beneficial, as they lessened some of the awkwardness that other participants often felt during face-to-face interviews when discussing private disease-related matters (i.e. dark urine or depigmented areas of skin on their genitals). In general, participants from both disease groups were open about their disease symptoms and eager to share their stories in living with and managing their conditions.

To be eligible for participation, all patients had to have received a formal diagnosis from a medical professional at the time of the interview, and they were also required to have lived in the US for a minimum of five years. All of the people that I interviewed with vitiligo were from the US and had only ever lived in that country, and all but three of the AKU patients were from, and had always resided in, the US. Of the three AKU patients who were not from or residing in the US at the time of the interview one was from the UK but living in the US, another was born in the US but moved to the UK at the age of fourteen, and the other migrated to the US from a small European country when she was in her mid-twenties. All of the interviews were transcribed verbatim by the author and analysed using a qualitative data processing program (MAXQDA 12). Initially, the author open coded each interview in order to determine any specific points of interest that individual participants detailed. The author then grouped any overlapping codes that emerged from the initial coding process into broader themes relating to the particular experiences of people with either disease, including: concerns about bodily fluid, concerns about appearance, difficulty in forming romantic attachments, feeling misunderstood by doctors, etc. From there, the author analysed these overlapping codes within wider themes concerning the lived experiences of people with chronic diseases more broadly, including: pain, passing, resilience, social/ economic inequality and hope. This study was given ethical approval by the internal review board at the author’s academic institution.

### How patients pass: mechanisms and techniques

When in public, AKU and vitiligo participants employed different strategies in order to pass as “healthy” or unaffected by their condition. Doing so allowed them to mitigate and, to an extent, control the amount of attention that their disease symptoms attracted from other people.^10^ These strategies often involved significant cosmetic and/ or lifestyle alterations, which varied in accordance with the participant’s disease stage. The more progressive the participant’s symptoms were the more difficult their symptoms were to conceal and, therefore, the less likely they were to pass successfully. In an effort to prove to family members, doctors and close friends that they were still able to participate in social activities that they enjoyed before their symptoms began to manifest, it was common for AKU patients to physically exert themselves by pushing their bodies beyond what they were physically capable of. This demonstrated a form of passing because it typically only took place whilst others were watching them and involved the patient concealing any evidence of their strain or difficulties in completing the task. Typically, these patients were aware of the fact that they would likely experience an increase in joint pain the following day, which would, in turn, likely leave them temporarily bed-ridden. However, for them, this pain was a small price to pay for the feeling of accomplishment that they would receive after completing these activities. This was particularly the case when patients viewed the activity as a central component of their identity. Engaging in these activities meant that they were able to retain a sense of who they were by continuing to do what they had previously enjoyed. Additionally, it enabled them to feel somewhat in control of a disease that continuously threatened to erode their capacity to “be themselves” in relation to how they self-identified.

During her interview one AKU patient who was diagnosed with kidney stones (a common AKU symptom) claimed that, after she underwent surgery to remove them, she “carried on as normal” by riding her horse the same day: “I went in to the hospital, got a stent taken out, they put me under anaesthetic and broke up the stone, put another stent in and I was riding my horse that afternoon at four o’clock.” Determined to maintain her daily routine and show her peers at her local riding stable that she was “fine,” this patient purposefully pushed her body beyond its limit in order to minimise the impact that her disease had on her lifestyle. By riding her horse the same day as her surgery, this patient was able to claim a semblance of normality whilst in the company of her peers by passing as physically capable when, at that moment, she was not. The pain that she endured as a result of pushing her body beyond its limit was, for her, worth the satisfaction of knowing that she was able to participate in an activity that she had always enjoyed.

Because vitiligo is a “cosmetic disease,” the participants that I spoke with were not concerned about the physical costs of over-exertion when attempting to pass as “healthy” or unaffected. However, participants routinely expressed their concerns about the emotional costs of concealing their disease with clothing or make up and the negative long-term effects that this had on their mental health. During their interviews, numerous participants discussed having experienced prolonged periods of depression as a result of failed attempts to conceal evidence of their disease in order to pass as “normal.” Many knew of other people with vitiligo who had attempted suicide because of their disease-related mental health issues. These participants would often discuss their concerns about the long-term impact of this emotional strain and the ways in which they understood it to negatively affect their physical health. They would also often relate their frustration with what seemed to them to be futile attempts to conceal a disease that not only changed their appearance but was also psychologically detrimental. As argued by one participant:

You can’t cover up something that’s not just physical but [also] psychological. It’s kind of like having a secret, no one knows when you have a secret of something that happened to you that you don’t want anyone to know, now you have something that’s so pronounced in the room that people see. You can’t hide it.

(Michelle, vitiligo)

For Michelle, even if she was successful in concealing her vitiligo and passing as “normal,” the psychological effects of the disease render it impossible to hide. Dark-skinned vitiligo participants are particularly vulnerable to everyday forms of negative attention and discrimination because the disease is significantly more visible on them than it is on white or light-skinned people who have it. For dark-skinned people who have not completely depigmented, their disease is immediately visible to others when it covers areas such as their face, neck and hands.^11^ White people with vitiligo, however, often have the advantage of being able to conceal their depigmented skin and regain a semblance of their “normal” appearance by applying mainstream tanning products that are readily available in drug stores. These products often make the white depigmented persons appear “naturally tanned” and “healthy” after they have applied them, and in doing so allow them to pass as “normal” or “unaffected” by the condition. As noted by one white participant:

Once it [vitiligo] pretty much took over my body I started wearing tanner … I wear tanner now because I'm like all one colour again [completely depigmented]. And so wearing tanner is pretty much like I'm free again like from everything. And I remember the first day I was wearing full tanner and I actually went into the little downtown area in my city and no one stared at me! And I was like oh my God I'm wearing an invisibility cloak right now, like no one can see me! This is so cool! I was so freaking excited I was like this is what it's like to be a normal person, this is amazing!

(Vanessa, vitiligo)

For Vanessa, wearing tanner means that she is able to socialise in public spaces without being seen in relation to her condition. The invisibility that she is afforded because of this product means that she feels “free” when in these spaces and is uninhibited by the stares and comments that often came before she completely depigmented. Because their skin colour is typically darker than the mainstream tanning products that Vanessa uses, dark-skinned people with this condition are not afforded this type of opportunity to become “invisible.” During her interview, Vanessa acknowledged this difference by arguing that, because they cannot conceal evidence of their disease by artificially tanning, black/dark-skinned people with vitiligo are more likely to seek relief by openly communicating with other people about their disease:

Pretty much every white girl I’ve talked to tries to hide it [vitiligo], and that’s like their go to way of coping with it. African-American people just like they have to talk about it. Like there’s not really another choice, they can’t really hide it the way lighter skinned people can. And I’ve often noticed that because of that there seems to be like a racial divide within the vitiligo community … I do think that there is just, I don’t know, there’s like a cultural difference about how you approach it. And I just get that feeling because like when you start meeting different pockets of people with vitiligo black people are always grouped together and like white people are always grouped together.

(Vanessa, vitiligo).3

According to Vanessa, racial divisions within the vitiligo community are the result of a mutual understanding of the ways in which this disease affects different groups in different ways. She infers that, because black people presumably share an understanding of what it means to live with this condition, they are naturally more inclined to socialise with other black people in the vitiligo community. In a similar way, because they too presumably share similar experiences, white people with vitiligo are more likely to socialise with each other at vitiligo events and community meetings. Thus, by interacting with other people from the same racial group, people with vitiligo feel more able to share their experiences of living with it and exchange information about cosmetic products that are particularly suited to their skin tone.

People with vitiligo who do not have white patches on areas of their body that are immediately visible to onlookers (such as their face or hands) are often able to pass as “normal” by wearing clothing that conceals affected areas of skin. Clothing that conceals these areas can be perceived as either intentional or inadvertent acts of passing depending on the individual circumstances surrounding the wearer’s choice of garment. It is often difficult to equivocally state whether persons with vitiligo who wear concealment clothing do so with the intention of passing or whether they pass unintentionally. Persons who have visibly depigmented areas of skin on their arm might purposefully wear long-sleeved shirts in order to conceal this from public view, or they might choose to wear them simply to stay warm. Whilst the former is premeditated, the latter is unintentional and does not demonstrate a purposeful attempt to pass as someone who does not have vitiligo. AKU patients often relate feelings of embarrassment and/ or shame when evidence of dark sweat and/ or dark urine is detected on their clothing. As previously stated, others often mistake both as evidence of a lack of hygiene rather than as symptoms of a genetic condition. As a result, many patients purposefully choose to wear dark clothing in order to minimise the visibility of these symptoms. Additionally, AKU patients often regularly replace their clothing when the dark stains become difficult to wash out. As noted by one patient:

When I was working we used to, the dress style was coat and tie and it was generally white shirts, and while the shirt was not worn out I would have to replace them sooner than I otherwise would have to. Because under the arms and around the neck it had a tinge on it, you know from the sweat. And they you know you can also see for example if we don’t replace the pillows frequently enough, that even you know just sleeping and drooling and whatever else goes on when you’re sleeping it’s the same thing. And so I know that all of my bodily fluids are dark and will stain and so forth so I don’t wear white underwear, just coloured underwear. I tend to wear more like this [points to shirt], this blue shirt and things like that so you really can’t see what’s going on. And so I mean you know it’s just, I’ve accommodated myself. I don’t wear any sort of white or light coloured pants because as guys do they sometimes don’t completely clean up after they urinate and so as a result you know instantly it stains. And if it’s a shirt tail then you can tuck it in but if it’s pants you know you get maybe 5 or 6 uses of them and then you have to throw them away and start over again with another pair.

(James, AKU)

James’s ability to pass as “healthy” or “unaffected” by his disease is made possible by his ability to minimise the visibility of his dark sweat and urine by wearing dark-coloured clothing. Before he retired James was a lawyer, and, whilst working in his office, he was obliged to wear white shirts that quickly made these symptoms visible. In order to conceal his symptoms from other people, he had to frequently replace them with new white shirts that were not yet stained. Additionally, at the time of the interview, James purposefully chose to buy dark-coloured pants that did not show dark-coloured sweat and urine stains. Whilst his reasoning suggests that this necessity is gendered (“as guys do they sometimes don’t completely clean up after they urinate”), during their interviews female AKU patients also discussed purposefully choosing clothing that minimised the visibility of their symptoms:

I wear a lot of black clothes. In my closet I have a lot, I wear maroon and black a lot. Maybe I’m being self-conscious because I’m a girl, I don’t know. But yeah I mean a lot of my shirts for exercising and stuff are darker. The ones that aren’t darker they turn a lot quicker you know underneath the arms and in the neck where I sweat. So that’s kind of embarrassing. And then I throw them away, white shirts I wear a couple of times then throw away. Unless I’m on Nitisinone, then they last a little longer. But it’s just part of the AKU, right?

(Claire, AKU)

For Claire and James, routinely replacing their clothing is a way of ensuring that their disease remains hidden from others, allowing them to pass as “normal” or “unaffected” by their condition. However, it is important to remember that AKU patients are only able to regularly replace their clothing if they have the financial means to do so. Patients who are unemployed and/ or financially dependent on the state are typically unable to regularly buy new clothing, which prevents them from concealing evidence of their disease in this way. For many patients who do not have the financial means to replace their clothing, their stained clothing functions as a constant reminder of their disease and its symptoms. Despite being able to attribute the stains to symptoms of a medical condition that they are unable to control, these patients are often made to feel personally responsible for them by others who understand these symptoms to be indicative of their “dirtiness” and/ or “uncleanliness.” As noted by Janet who, at the time of the interview, was unemployed and waiting to hear the outcome of her application for disability benefits:

When you have AKU you think oh well you’re kind of different. You’re dirty [and] you have all this stuff that’s gross and your urine smells more than other peoples’ and your sweat leaves dark marks. Because I can’t afford new underwear I’m stuck with the same bras and they always show the marks.

(Janet, AKU)

Because Janet could not afford to replace her clothing, which showed visible signs of her dark sweat, she was constantly aware of it in a way that patients like Claire and James who could afford to regularly replace their clothing were not. In other words, due to her relative poverty, Janet was unable to pass as “normal” in the same way that they could because her dark sweat remained visible. However, when asked about her attempts to conceal some of her other disease symptoms Janet responded by saying that her affected gait often went unnoticed when she was in public spaces if she was with her dog. The dog, she claimed, would often become the sole point of focus and would thus draw other people’s attention away from her “bad” posture, rendering her visible differences invisible. That, combined with the physical stability that her shopping cart gave her when she was shopping for groceries, enabled her to pass as “normal” when in public spaces such as her local supermarket:

If I have the dog with me the dog is so cute that everybody goes ‘oh look at the dog’ and I think she’s like a good buffer, you know? I can push the cart at the grocery market, I can push the cart and I don’t look like anything’s wrong. That’s why I think I need a walker that’s up here [gestures high], because if I just [peers over top of imaginary cart] around the market I don’t look any different. It might be you’re just going and following the cart and then my dog is right there and they’ll look at her and not me.

(Janet, AKU)

By deflecting attention away from her bodily differences, both her dog and the cart allow Janet to pass as “normal” and, thus, lessen her self-consciousness about being seen in relation to her symptoms and her condition.

### Wearing a mask/ being in the closet

It felt nice to take the mask [make up] off because when you put the make up on you’re hiding behind someone that you’re not, and when you’re hiding that person you’re also hurting that person. So I just felt like I was pretending to be you know like a full caramel person [skin colour] when I’m really not, you know? I have spots on my body and so why should I cover them? And so it took me a really long time to just be like do you know what, I’m not going to spend this extra 20 minutes on my face right now to try and cover up this white spot right here. I’m just going to leave it as it is.

(Lauren, vitiligo)

I never bring it [AKU] up but if somebody asks and is interested I will talk to people about it, you know? I’ve learned kind of with how people are to kind of keep it quiet if you will, because you get different people just saying different things or not knowing what you’re talking about. And like I said what I ran into the other day just little scenarios of that where one person knows a little bit more than another, or they think they know a little bit more than the other, and they don’t grasp really what it is but they’re like spouting out stuff. So I want the knowledge to be out there but after some comments on messages that I’ve posted on social media I usually am like ok, I better take this post down now. I’m in the closet, I’m kind of in the closet I feel. Like I feel like I kind of have to hide my AKU. Only if somebody really wants to know do I sit there and tell them about it … like I can understand and relate to people that are like in the closet about anything or everything, I can you know relate to that because it’s something I feel like I have to hide. I don’t want to hide but I do, you know?

(Claire, AKU)

I remember when, it sounds funny, when I “came out” at church [laughs]. You know like I wore the shorter dress and I had my arms out and we marched for offering at church and I remember carrying my goddaughter and when I got back to my seat and church was over people were like ‘oh I didn’t know you were sick,’ that’s when the albino business came in. I finally was like you know what it is too hot for this [long-sleeved clothing to cover vitiligo] and it is too hot for that. This is, you know, me - hello! See me.

(Elizabeth, vitiligo)

These excerpts convey typical reactions from participants from both disease groups who, when asked why they choose to reveal or conceal evidence of, or information about, their disease, claim that their decision originates from experiences that they have had with people who respond negatively to them because of it. For Lauren, efforts that she had previously made to pass by covering her vitiligo with make-up made her feel as though she was “pretending” to be someone she is not (i.e. a person without vitiligo). Whilst passing made it possible for her to avoid stares and comments from other people, it also prevented her from expressing who she is in relation to how she sees herself, which negatively affected her mental health. Lauren’s decision to remove the “mask” by no longer wearing make-up demonstrates a conscious effort to prioritise her mental health above the negative attention that she received from others because of her appearance. By showing her vitiligo-marked skin, Lauren no longer felt that she was “hiding” an identity that she was later able to proudly claim as her own. For Claire, the fear of being misperceived and/ or misunderstood when talking about AKU is a key motivator for concealing all knowledge of it. In order for her to disclose details of her disease, she first needs to be invited to do so, and she then only reveals a minimal amount because she fears that the listener will misunderstand what she is saying and will later inaccurately relate the information that she has given them to other people. Knowing that others falsely believe that her disease affects her in ways that it does not causes Claire a great deal of anxiety, and this motivates her to remain “in the closet.”

For Elizabeth, her decision to “come out” by showing her vitiligo whilst at church was the result of a practical feeling of being “too hot” in clothes that hid her condition from view. After she revealed her vitiligo she was presumed to be “sick” by other members of the congregation who mistook her symptoms as signs of poor health. She later corrected them by outlining what the disease was, what it meant for her, and how it differed from albinism.^12^ The discrepancy between Lauren’s view that passing is detrimental to her emotional wellbeing and Claire’s view that passing is necessary in order to ensure hers is influenced by the degree to which both are able to *choose* whether or not to pass. Because Lauren’s condition is difficult to conceal in all areas of her body, it is always immediately visible to onlookers.^13^ Moreover, she cannot choose to pass in ways that render her condition wholly invisible, which means that, for her, learning to accept how she looks with vitiligo involves learning to accept how other people see her. For Lauren, this acceptance is key to building resilience against the daily stares and comments that she receives that constantly remind her of her visible differences. Because AKU does not mark her body in any visible way, Claire can choose not to disclose information of it and, thus, can choose to avoid any negative attention that it might bring. Whilst this discrepancy suggests that Claire’s ability to choose when to reveal or conceal her disease places her in a favourable position, it also means that, unlike Lauren, Claire has to explain the fact that she has a disease to other people and, in some cases, prove its existence in order to be seen in relation to it.^14^ Her silence prevents others from recognising how her everyday symptoms (i.e. chronic pain, dark urine and dark sweat) impact her life in meaningful ways, which makes her feel as though she is “in the closet.”

The contention between choosing when to pass and when to disclose is one that is central to all traditional passing narratives. Numerous black people who passed as white during the Jim Crow era chose to do so at various intervals in order to profit from employment opportunities that they otherwise would not have had. Whilst they passed as white at work they often returned to their families and reclaimed their black identities at the end of the day, a phenomenon that passing historian Allyson Hobbs refers to as “working white while living black” (2014). Knowing that they were returning to a space that would allow them to recover their racial identities meant that they were able to withstand many of the psychologically damaging effects of passing. Common effects included experiencing an identity crisis and permanently disowning close friends and family members (Hobbs 2014). On days when they feel particularly conscious of their appearance, people with vitiligo often choose to remain in their homes either by themselves or accompanied by family members. For many, the support of their families and the comfort of being in a familiar space temporarily relieves the burden of “looking different” and allows them to go about their daily lives without being constantly reminded of their disease and the ways in which it makes them “different.” In this way, participants are able to pass as “normal”/ unaffected by their condition whilst they are at home. As noted by one participant:

Everybody would be encouraging me to go out and do things and I’d just be like no, I don’t want to do it because I don’t want to go out there and have to deal with the stares and the looks and everything. I’m just like so sick and tired of it. I’m like you know what I don’t want to. I don’t want to deal with that today. I don’t want to acknowledge the fact that I have vitiligo and I look different than everyone else, I just want to be inside chilling in the house and watching TV or doing nothing or playing video games. Or you know I’ll wait until night time and just go to a movie theatre where everything is dark in there and no one’s staring at me because everybody’s watching the movie or whatever.

(Anthony, vitiligo)

Anthony’s anticipation of the negative responses that his appearance would invite when he entered public spaces prompted extended periods of social isolation. During this time, he would purposefully avoid social spaces in favour of remaining at home where he could, temporarily, forget about his vitiligo. Viewing his home as a safe space, Anthony felt able to carry out everyday activities without being consciously aware of his body and how it “differs.” However, unlike Anthony, numerous other vitiligo participants who lived with family members often attempted to hide their condition whilst at home by wearing make-up to conceal it. Rather than viewing their home as a safe space, these participants understood it as a place that rendered them vulnerable to negative remarks from those who were closest to them. In this way, their attempts to pass whilst at home serve as a recognition of the fact that these spaces are “unsafe” whilst they are in them and make up free. They are viewed as particularly unsafe when people from outside of their immediate family visit their home. During her interview, one participant discussed how, when her children were young and had friends sleep over at her house, she would go to bed late at night and wake up early in the morning in order to ensure that her children’s friends did not see her without make up:

In my house yeah my family they would see me without make up but … when my kids had other kids stay over or whatever what I would do was I would make sure you know that they were you know fed or they had their little snacks or whatever they needed and after that I would wash my face and stuff and I wouldn’t go back out [leave bedroom] no more. So really 9.30/ 10 that was it, I didn’t come back out again until the morning. I would get up before they would, I’d get up probably 6 or 7 o’clock before anybody. I’d have been and already took my shower, I’m already completely already dressed and got my make up on and everything. So right now the girls that come over that stay all night with my daughter they be like ‘when did your mom’s face get like that? I didn’t know her face was like that’ because I put it [make up] on before they woke up when they were children they never knew I had it [vitiligo].

(Sarah, vitiligo)

When her children did not have visitors, Sarah viewed her home as a “safe space” in which she could walk around without make-up and not be criticised. Once she got home, she would routinely “remove the mask” of make-up that she had applied earlier that morning and allow her children to see her with vitiligo. However, when other children visited her home, she viewed it as temporarily “unsafe,” as these children could potentially criticise her because of her appearance whilst they were there. Fearing this, Sarah kept her make up on when they were in her home until she was sure that they would not see her. In doing so she was able pass as a “normal” when around them, and her success is made evident by the surprise that these children showed years later upon discovering that she had had vitiligo for decades (“they never knew I had it”). On occasion, Sarah would be caught off-guard by the sudden appearance of these children in her home, and, because her children had failed to pre-warn her, she would react negatively towards them:

Sometimes when I came out the living room thinking it was only us [family] in the house some of my kids friends were there and I would, I’m not going to lie, sometimes I would just start yelling and stuff and go back in my room and stuff like that. I’d be like ‘why didn’t you tell me somebody was in here?!’ [aggressive tone] and stuff like that. I’d say ‘you see how my face is’ and stuff like that so [starts crying] I think about that stuff. I know they [her own children] understand now you know but when I just look back at it and see myself and the way that I was reacting and the stuff that I said I feel bad because that was probably hurtful for them.

(Sarah, vitiligo)

By inviting their friends into the house without warning her, Sarah’s children unintentionally exposed her to a type of harm that she only anticipated when she left her house. Unlike Lauren who was able to decide when to make her vitiligo known, Sarah was prevented from deciding whether to “come out” as a person with vitiligo because her children unknowingly made that decision for her by inviting their friends to visit. It was only after Sarah accepted her disease and embraced the changes that it had made to her appearance that she could understand the damaging effects that her aggressive responses had on her children.

Like people with vitiligo, AKU patients often claim that, for them, their homes function as “safe spaces” because they offer protection from disease-related stares and comments. Because AKU is a rare condition, it is common for people to be unfamiliar with it and to mistake visible symptoms of the disease for signs of other conditions or “deviant” behaviour. Because their gaits are commonly affected, older AKU patients often stumble when in public spaces and, as a result, are often mistaken for alcoholics or drug addicts. As noted by one AKU patient, “I walk like a goofy person, sometimes like a penguin. You might think I’m drunk or you might think [I’m] pretending to walk like this.” By remaining at home, patients are able to avoid these mischaracterisations that invariably lead to stigmatisation (Goffman 1990, 14).

In addition, AKU patients often view their homes as “safe” because they have practically adapted them to meet their mobility needs. Older patients, in particular, often claimed that they purposefully modified their homes in order to meet their current and future mobility needs and that this relieved some of their concerns about falling and causing further joint damage when they were at home. By equipping their homes to suit their needs, AKU patients felt able to temporarily overlook the limitations that their disease symptoms imposed on them. In this way, whilst they were at home they were able to somewhat pass as “healthy” or “able-bodied” because they were free to carry out ordinary tasks with the help of these aids. It is, however, important to note that, for some patients, their homes do not function as “safe” spaces because they allow others to see “embarrassing” symptoms on a regular basis. When relating how she dealt with having dark urine as a child, one patient discussed how she was regularly self-conscious about it and that this was exacerbated by her mother’s negative remarks about the stains she would leave on her clothing after urinating:

I was self-conscious about my urine when I was little because it turns black. Because it turns black I would be very self-conscious about it and so like say one time my mom made a joke but I didn’t know it was a joke. I go can I go to the bathroom? And she goes ‘no’ and I go why not? And she says ‘oh because they’re working on the sewer down the street’ and she makes up this story that anybody who flushes the toilet it’s gonna go and everybody’s going to see it you know or whatever and they’re gonna know it was you because it turns black. And so anyway so I was self-conscious always. So I would like I could hear other people in my house, my mom didn’t realise I believed her I guess because I could hear other people in my house going to the bathroom and my brother and my mother and my father they would all go and just go and then leave the room like nothing. They weren’t even sneaking. And then I would have to get up early and sneak and go in there and pee. Or go pee outside and then dump water over it.

(Janet, AKU)

The necessity that Janet felt to “sneak” to her bathroom early in the morning was the direct result of the uneasiness that she felt in having other members of her family see her dark urine. Later in the interview, Janet discussed the fact that she continues to feel shame about having this symptom as a result of these negative remarks and that this shame is linked to her understanding that having dark urine is a sign of uncleanliness Her attempts to hide evidence of her dark urine from her family members demonstrates a desire to pass as unaffected by this symptom whilst at home, which is something that she was often unable to do.

## Conclusion

As this article has shown, the social and financial means of patients from both disease groups largely determines their ability to effectively pass as “healthy” or unaffected by their condition, and access treatments that minimise the visibility of their disease symptoms. AKU patients who have access to Nitisinone experience a reduction in the accumulation of homogentisic acid, which, in turn, means that they are less likely to suffer from chronic pain and mobility issues later in life.^15^ This, then, means that they are more likely to pass as “normal” or “unaffected” by their condition than those who do not have access to this drug. In addition, these patients are less likely to require joint replacement surgeries as they age and their symptoms progress. In a similar way, people with vitiligo who are able to afford costly treatments that are not covered by medical insurers are more likely to show signs of repigmentation over the course of their disease (Rodrigues et al. 2017). Effective treatment options often require a financial commitment from those affected, many of whom are likely to be, at best, only partially subsidised for them by medical insurers. Thus, in both disease groups, the patient’s ability to pass is largely dependent on their ability to financially cover the costs of treatments that will minimise their disease’s visibility.

The decision that patients make to pass is also largely dependent on their disease stage. If their symptoms have advanced to the point where they can no longer be concealed, patients are unable to easily pass as “healthy” or “unaffected.” Because AKU and vitiligo are largely unknown diseases, it is easy for onlookers to misinterpret their symptoms as signs of other health conditions. In anticipation of this, patients often wear signs that visibly mark them as having AKU or vitiligo. For vitiligo patients, these signs typically come in the form of badges that reassure onlookers that their disease is not contagious. These badges commonly include statements such as “if you can wear your tattoos, I can wear my skin” that highlight the harmlessness of their condition. In doing so, they resist the often immediate assumption that patients should be pitied or feared because of their vitiligo. Moreover, by comparing their disease to elective forms of skin modification (i.e. tattoos), these patients signal the fact that, for many, having vitiligo is an aesthetic marker of uniqueness rather than a stigmatising signifier of difference.

Similarly, in an effort to increase awareness about the prevalence of invisible diseases, AKU patients often display stickers on their car that include phrases such as “not every disease is visible” to make their disability known. By displaying them on their car, patients hope to avoid confrontation when publically parking in areas that are purposefully reserved for disabled motorists. Although they do not directly state their condition and its symptoms, these stickers allow AKU patients a degree of autonomy by purposefully revealing their disability status to unsuspecting onlookers. By providing visual clarification and confirmation of the nature of both conditions, these signs allow patients to correct misperceptions that often result in stigmatisation. They also offer patients the opportunity to proudly claim their disease in a public way and render passing an unnecessary practice. Future discussions about passing in a disease/ illness context should further consider the ways in which resistance strategies are implemented by patients as a way to reclaim and/or change stigmatising narratives about (in)visible diseases. This seems particularly salient in an era where social media platforms are routinely being used to explore what it means to be “well” and/or “look healthy” despite a steady increase in the number of people being diagnosed worldwide with chronic conditions (Stuckler and Siegel 2011; Mercer 2014; De Maio 2014).

## Endnotes

^1^ For further information see Scales-Trent 2001.

^2^ Here I use the term “normative identity categories” to describe primary identity categories (i.e. race, gender and class) that are typically assigned to people based on what their bodies seemingly suggest about them.

^3^ For further information see Sanders et al. 2002.

^4^ Here I have chosen to use quotation marks when describing people from both disease groups as patients because some of the people that I interviewed with vitiligo were resistant to this label.

^5^ Note that there is currently no cure for vitiligo. For further information see: Porter et al. 1979,

^6^ These symptoms typically occur after patients reach the age of 30.

^7^ Whilst these symptoms are largely invisible to unsuspecting onlookers, it is possible for medical professionals who have heard of AKU to recognise these symptoms as signs of the condition.

^8^ In relation to chronic pain and fatigue.

^9^ Note that I interviewed one US patient in the UK and spoke with two other UK-based patients about their experiences using the National Health Service (NHS) in order to consider any interesting and/or relevant connections between the two countries.

^10^ Note that participants are limited in their ability to control the amount of attention that their disease symptoms attract because they cannot control the individual subjectivities of the onlookers who criticize them.

^11^ Participants reported feeling particularly self-conscious when their vitiligo spread to these areas because they are immediately visible to onlookers.

^12^ There is a common misconception that vitiligo is directly linked to albinism as both result in a loss of skin pigmentation.

^13^ This is, of course, largely dependent on the clothing that she wears.

^14^ During her interview Claire described at length how she was often required to prove that she had a medical condition to others who, because they had not heard of AKU, did not believe her.

^15^ Note that joint pain derives from a build up of homogentisic acid, which causes ochronotic pigment to form on the patient’s bones and joint cartilage, eventually leading to joint and bone deterioration and, often, chronic pain.
